# Comparing Cancer Risks and Mortality between Phytopharmaceuticals and Estrogen-Progestogen Medications for Menopausal Women: A Population-Based Cohort Study

**DOI:** 10.3390/healthcare12121220

**Published:** 2024-06-19

**Authors:** Tsai-Bei Lin, Chia-Chi Hsieh, Chun-Hsiang Wang, Chiung-Hung Chang, Yu-Ling Hsueh, Yuan-Tsung Tseng, Men-Fong Hsieh

**Affiliations:** 1 Department of Obstetrics and Gynecology, Bao Hua Tang Traditional Chinese Medicine Clinic, Tainan 701033, Taiwan; linemailtmh@yahoo.com.tw; 2 Departments of Nursing, Chang Bing Show Chwan Memorial Hospital, Changhua 505029, Taiwan; 3 Departments of Nursing, Show Chwan Memorial Hospital, Changhua City 500009, Taiwan; 4 Department of Hepatogastroenterology, Tainan Municipal Hospital (Managed By Show Chwan Medical Care Corporation), Tainan 701033, Taiwan; 5 Department of Traditional Chinese Medicine, Tainan Municipal Hospital (Managed By Show Chwan Medical Care Corporation), Tainan 701033, Taiwan; 6 Department of Obstetrics and Gynecology, Tainan Municipal Hospital (Managed By Show Chwan Medical Care Corporation), Tainan 701033, Taiwan; 7 Department of Medical Research, Tainan Municipal Hospital (Managed By Show Chwan Medical Care Corporation), Tainan 701033, Taiwan; b891040733@yahoo.com.tw

**Keywords:** menopause, phytopharmaceuticals, hormone replacement therapy, overall cancer, all-cause mortality, National Health Insurance Research Database

## Abstract

We evaluated the long-term risks of overall cancer and all-cause mortality associated with five types of phytopharmaceuticals and the most commonly used estrogen-progestogen medications for the treatment of postmenopausal syndrome in women. Using data from Taiwan’s National Health Insurance Research Database (NHIRD) from 1 January 2000 to 31 December 2018, we conducted a 1:2 matched cohort study with 12,087 eligible patients. We compared phytopharmaceuticals -only users (n = 4029, phytopharmaceuticals group) with HRT-only users (n = 8058, HRT group) with a washout period of ≥6 months. The phytopharmaceuticals group had significantly lower risks of overall cancer and all-cause mortality than the HRT group (adjusted hazard ratio [95% confidence interval]: 0.60 [0.40–0.9] and 0.40 [0.16–0.99], respectively) after over 180 days of use. Bupleurum and Peony Formula were associated with lower risks of overall cancer and all-cause mortality (aHR: 0.57 [0.36–0.92] and 0.33 [0.11–1.05], respectively). In conclusion, phytopharmaceuticals may serve as an alternative therapy to HRT for alleviating menopausal symptoms and reducing health risks, leading to more favorable long-term health outcomes. Further randomized control trials are necessary to validate the findings of this study.

## 1. Introduction

Prior to the Women’s Health Initiative (WHI) study, hormone replacement therapy (HRT) was widely used to treat menopausal syndrome. However, the WHI study and other studies have indicated that HRT is associated with increased risks of coronary heart disease, stroke, and venous thromboembolic disease, regardless of the duration of therapy, bringing certain health risks [1,2,3].

Menopausal syndrome is a common health issue affecting women worldwide, with a prevalence of up to 50% among Taiwanese women aged 40–60 years [4]. Effective management of these symptoms is crucial to improving the quality of life for affected women [5].

Various treatment options are available for managing menopausal syndrome, including pharmacological treatments such as HRT and non-pharmacological treatments such as lifestyle modifications, alternative therapies, and herbal medicines [6]. Pharmacological treatments primarily include HRT, which involves the administration of estrogen and progesterone to alleviate symptoms. Non-pharmacological treatments include lifestyle modifications such as maintaining a healthy diet, regular physical activity, and stress management techniques like mindfulness and yoga, which can help reduce the severity of symptoms [7]. Additionally, alternative therapies such as acupuncture and massage therapy have shown potential benefits in symptom relief [8].

Herbal medicines, or phytopharmaceuticals, derived from plants and used in traditional medicine, offer another avenue for managing menopausal symptoms [9]. The main phytopharmaceutical formulas investigated in this study, along with their composition and therapeutic effects, are summarized in Supplementary Table S1. These formulas, including Bupleurum and Peony Formula, Anemarrhena Phellodendron and Rehmannia Formula, Ginseng and Zizyphus Formula, Zizyphus Combination, and Magnolia and Ginger Formula, have been widely used in clinical practice to improve menopausal symptoms. Since 1995, Taiwan’s National Health Insurance program has been covering both HRT and phytopharmaceutical medicines [10]. Several studies have investigated the efficacy of phytopharmaceuticals in managing menopausal symptoms, but few have comprehensively compared their long-term safety with HRT. There is a need for more research to evaluate the long-term risks associated with these treatments, including overall cancer risk and all-cause mortality.

In this study, we compared the long-term risks of overall cancer and all-cause mortality associated with HRT and phytopharmaceuticals for menopausal syndrome in Taiwanese women using a large real-world dataset with a long follow-up period and a new-user design to balance baseline characteristics.

## 2. Materials and Methods

### 2.1. Data Source

For this longitudinal population-based study, we used data from Taiwan’s National Health Insurance Research Database (NHIRD), which includes the claims data of over 99% of Taiwan’s population, reflecting the demographic and health characteristics of the general population [11]. As a result, findings from the NHIRD can be considered representative of the Taiwanese population. We retrieved all medical data, data on operations, and prescription history from 1 January 2000 to 31 December 2018. Diagnoses were identified using International Classification of Disease, Ninth Revision, Clinical Modification (ICD-9-CM) and International Classification of Disease, Tenth Revision, Clinical Modification (ICD-10-CM) codes.

The Research Ethics Committee of Show Chwan Memorial Hospital in Changhua City, Taiwan, approved the study protocol on 19 August 2020 (IRB No: 1080703) and waived the need for informed consent because all NHIRD data are encrypted secondary data, precluding participant identification.

### 2.2. Study Design and Study Participants

We considered phytopharmaceuticals and HRT as valid treatment initiations for new-user design patients, with a washout period of at least 6 months. Patients with menopause prescribed phytopharmaceuticals without HRT were assigned to the phytopharmaceuticals group, whereas those who received HRT without phytopharmaceuticals were assigned to the HRT group. We initially screened the top five phytopharmaceuticals most commonly used by menopausal patients in Taiwan that have received regulatory approval from the Taiwan Food and Drug Administration. The clinical efficacies of these formulations are supported by relevant literature, as summarized in the Supplementary Table S1. Phytopharmaceuticals included the following formulations: Bupleurum and Peony Formula (BPF), Anemarrhena Phellodendron and Rehmannia Formula (APR), Ginseng and Zizyphus Formula (GZF), Zizyphus Combination (ZC), Magnolia and Ginger Formula (MGF), Lycium Chrysanthemum and Rehmannia Formula, Bupleurum and Dragon Bone Combination, Licorice Combination, Rehmannia Six Formula, and Cyathula and Rehmannia Formula. HRT included the following prescriptions: estrogens (conjugated estrogen and estradiol) and progestins (progesterone, norethindrone, cyproterone, ethisterone, medroxyprogesterone, and norgestrel).

To assess the potential dose-response effect of phytopharmaceuticals on cancer risk, we conducted a sensitivity analysis. The follow-up period was divided into three categories based on the total number of days the prescriptions were taken, counted from the initial prescription date: 1–90 days, 91–180 days, and over 180 days. This allowed us to examine whether the duration of phytopharmaceutical use influenced the likelihood of cancer diagnosis.

### 2.3. Potential Confounding Factors

The exclusion criteria for patients were as follows: age < 50 years, diagnosis of cancer before the index date, a follow-up period of less than one year, and not meeting the washout period criterion of at least six months for the use of phytopharmaceuticals and hormone replacement therapy (HRT) prior to the index date.

The patients in the phytopharmaceuticals and HRT groups were matched at a 1:2 ratio by propensity score matching for the following variables: exact age; sex; Charlson comorbidity index (CCI); comorbidities, including diabetes mellitus (DM), hypertensive cardiovascular disease (HCD), and hyperlipidemia; and the index year (i.e., the year of the index date).

The final matched cohort consisted of 12,087 patients who were evaluated from the index date until cancer diagnosis, death, or 31 December 2018, whichever occurred first.

### 2.4. Covariate Measurements

To account for confounding factors, we adjusted for covariates potentially affecting the relationship between phytopharmaceutical use and hormone replacement therapy (HRT). These covariates included menopause (ICD-9: 627.x; ICD-10: N95), diabetes mellitus (DM; ICD-9: 250; ICD-10: E10-E11 series), hypertension (HCD; ICD-9: 401–405; ICD-10: I10-I15), and hyperlipidemia (ICD-9: 272; ICD-10: E78).

### 2.5. Primary Outcome Measures

Our primary outcomes were categorized into various cancer types and all-cause mortality, identified through specific ICD-9 and ICD-10 codes. The categories included ovarian (ICD-9: 183; ICD-10: C56, C570–C574), breast (ICD-9: 174; ICD-10: C50), endometrial (ICD-9: 182; ICD-10: C54), cervical (ICD-9: 180; ICD-10: C53), oral (ICD-9: 140–149; ICD-10: C00–C14), liver (ICD-9: 155; ICD-10: C22), colon (ICD-9: 153; ICD-10: C18), rectal (ICD-9: 154; ICD-10: C19–C21), thyroid (ICD-9: 193; ICD-10: C73), skin (ICD-9: 172–173; ICD-10: C44), lung (ICD-9: 162; ICD-10: C33, C34), gastric (ICD-9: 151; ICD-10: C16), renal (ICD-9: 189; ICD-10: C65, C66, C68), and bladder cancers (ICD-9: 188; ICD-10: C67), alongside an overall cancer classification (ICD-9: 140–208; ICD-10: C00–C96). We also monitored all-cause mortality up to the end of the study period.

### 2.6. Exposure Definition and Follow-Up

To mitigate the influence of extraneous factors, a washout period of six months was implemented for patients newly diagnosed with cancer [12]. The index date was defined as the first prescription date of HRT or phytopharmaceuticals for menopausal symptoms.

We recorded the prescriptions, main outcomes of cancer, and deaths from the index date until 31 December 2018. Therefore, the follow-up duration was from the index date to the first outcome date of cancer diagnosis, death, or 31 December 2018.

### 2.7. Statistical Analysis

We used the chi-square test to compare categorical variables and the t-test to compare continuous variables. Propensity score matching (PSM) was applied to minimize the potential selection bias, for example, from comorbidities, between the HRT and phytopharmaceutical cohorts. For matching based on the propensity score with a robust estimator, we adopted full matching without replacement. Subsequently, we employed a population regression model that appropriately accounted for the relationship between the outcome variables, treatment, and all covariates utilized [13,14]. Propensity score matching was performed through multivariate logistic regression and nearest-neighbor matching techniques, utilizing the “MatchIt” R package, version 4.3.4.

To estimate the stability and robustness based on hazard ratios (HRs) and their 95% confidence intervals (CIs), we used the bootstrapping method to modify the Cox proportional hazards regression models for determining the association between the risk of cancer and the target outcome between phytopharmaceuticals and HRT [15,16]. The cumulative incidence of mortality was estimated using the Kaplan–Meier method and was compared using the log-rank test. The Fine and Gray model was used to estimate the cumulative competing risks of death in each cancer. Sensitivity analysis was performed by evaluating the differences in curves by using both the log-rank test and the Fine and Gray model [17].

Adjustments for competing risks of death were made using the R packages “cmprsk” (version 2.2–11) and “mstate” (version 0.3.2). All statistical analyses were performed using SPSS version 21.0 (SPSS, IBM; Chicago, IL, USA) and R version 4.2.2 (R Core Team, Vienna, Austria). *p* < 0.05 was considered statistically significant.

## 3. Results

### 3.1. Patient Characteristics

**Figure 1:**
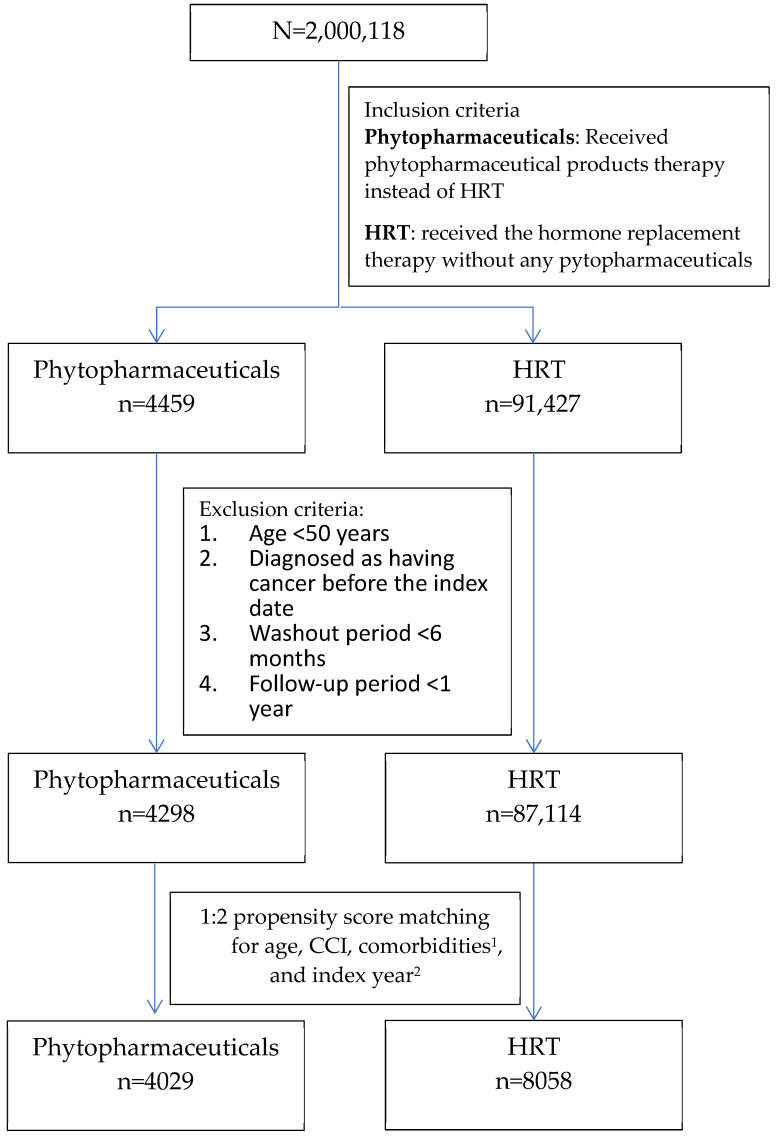
Flowchart of patient selection. ^1^ Comorbidities include DM, HCD, hyperlipidemia, and CCI. ^2^ Index year: exact year of diagnosis. Abbreviations used in this study: CCI—A measure of overall comorbidity burden, the Charlson Comorbidity Index. DM—Diagnosis of diabetes mellitus, HCD—Diagnosis of hypertensive cardiovascular disease.

From the NHIRD, we initially identified 4459 phytopharmaceuticals users and 91,427 HRT users during the study period (Figure 1). After the exclusion criteria were applied, 4298 and 87,114 patients remained in the phytopharmaceuticals and HRT cohorts, respectively, and after 1:2 PSM, 4029 and 8058 patients, respectively, were included in the analysis. Figure 1 presents the patient selection flowchart. The HRT and phytopharmaceuticals groups were fully matched by age, CCI scores, and comorbidities, including DM, HCD, and hyperlipidemia (Table 1). The baseline characteristics did not show any significant between-group differences, indicating adequate matching. The mean follow-up period was 8.69 ± 4.63 (median: 8.16) years for the phytopharmaceuticals cohort and 8.65 ± 4.68 (median: 8.17) years for the HRT cohort.

### 3.2. Comparison of Each Cancer Prevalence and Mortality Rate between Phytopharmaceuticals and HRT

Table 2 details the incidence rates for each type of cancer, overall cancer, and mortality rates. The phytopharmaceuticals group exhibited a lower incidence rate of overall cancer at 5.62 per 1000 person-years compared to 6.48 per 1000 person-years in the HRT group. Additionally, the rates per 1000 person-years for specific cancers were also lower in the phytopharmaceuticals group: endometrial cancer (0.17 vs. 0.96) and cervical cancer (0.20 vs. 0.72).

Table 3 reveals that the incidence of endometrial cancer was significantly lower in the group using phytopharmaceuticals compared to those on hormone replacement therapy (HRT) (0.1% vs. 0.8%; *p* < 0.001). This trend was also seen in cervical cancer rates (0.2% vs. 0.6%; *p* < 0.001). The overall cancer incidence and mortality rate were also lower in the phytopharmaceutical group, though not statistically significant (4.9% vs. 5.6%; *p* = 0.095 and 1.9% vs. 2.1%; *p* = 0.29, respectively). Further analysis using Cox regression showed that phytopharmaceuticals were associated with a reduced risk of developing endometrial cancer (HR: 0.17, 95% CI: 0.07–0.38; *p* < 0.001) and cervical cancer (HR: 0.32, 95% CI: 0.14–0.71; *p* = 0.005) compared to HRT. However, no significant links were found between phytopharmaceutical use and the other 13 types of cancer examined.

**Figure 2:**
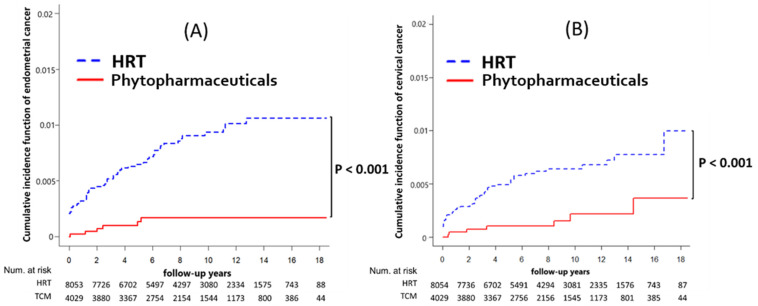
(**A**) Cumulative incidence of endometrial cancer between HRT and phytopharmaceuticals users after adjustment for competing mortality risks. (**B**) Cumulative incidence of cervical cancer between HRT and phytopharmaceuticals after adjustment for competing mortality risks.

**Figure 3:**
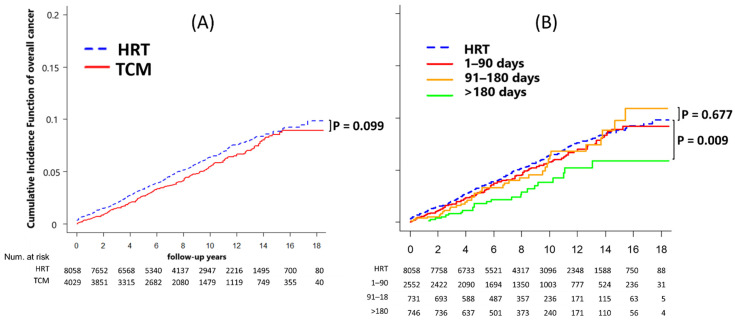
(**A**) Cumulative incidence of overall cancer between HRT and phytopharmaceutical users after adjustment for competing mortality risks. (**B**) Cumulative incidence of overall cancer between HRT and phytopharmaceutical users for different prescription durations after adjustment for competing mortality risks.

**Figure 4:**
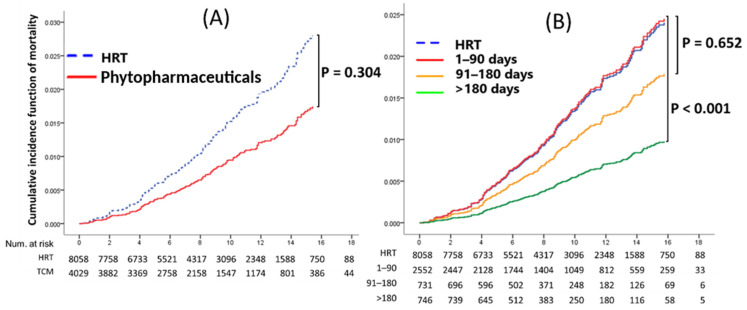
(**A**) Cumulative incidence of all-cause mortality between phytopharmaceuticals and HRT users after adjustment for competing mortality risks. (**B**) Cumulative incidence of all-cause mortality between HRT and phytopharmaceuticals for different prescription durations.

In the Fine and Gray competing risk model, the phytopharmaceuticals group had significantly lower risks of endometrial cancer and cervical cancer (both *p* < 0.001) than the HRT group (Figure 2). Furthermore, there were no significant differences in the rates of overall cancer (*p* = 0.099) and all-cause mortality (*p* = 0.304) between the phytopharmaceuticals group and the HRT cohort (Figure 3A and Figure 4A).

### 3.3. Phytopharmaceuticals Duration Effect on Cancer Development and Mortality

Phytopharmaceutical users were divided into groups according to drug exposure: 1–90 days, 91–180 days, and >180 days. For patients with >180 days of drug exposure, multivariate Cox regression analysis revealed that after adjustment for potential confounders, the incidence of overall cancer and all-cause mortality was 0.60 (95% CI: 0.40–0.90; *p* = 0.015) and 0.40 (95% CI: 0.16–0.99; *p* = 0.047) in the phytopharmaceuticals group compared with that in the HRT group (Table 4). We observed that some long-term phytopharmaceutical users had decreased risks of overall cancer and mortality.

As presented in Figure 3B, compared with patients with limited exposure to phytopharmaceuticals (1–90 days or 91–180 days) or those undergoing HRT, phytopharmaceuticals use for >180 days (*p* = 0.009) significantly decreased the risk of overall cancer. Furthermore, phytopharmaceutical use for >180 days was significantly associated with all-cause mortality (Figure 4B).

### 3.4. Effect of Top Five Phytopharmaceuticals Prescriptions on Cancer Development and Mortality

**Figure 5:**
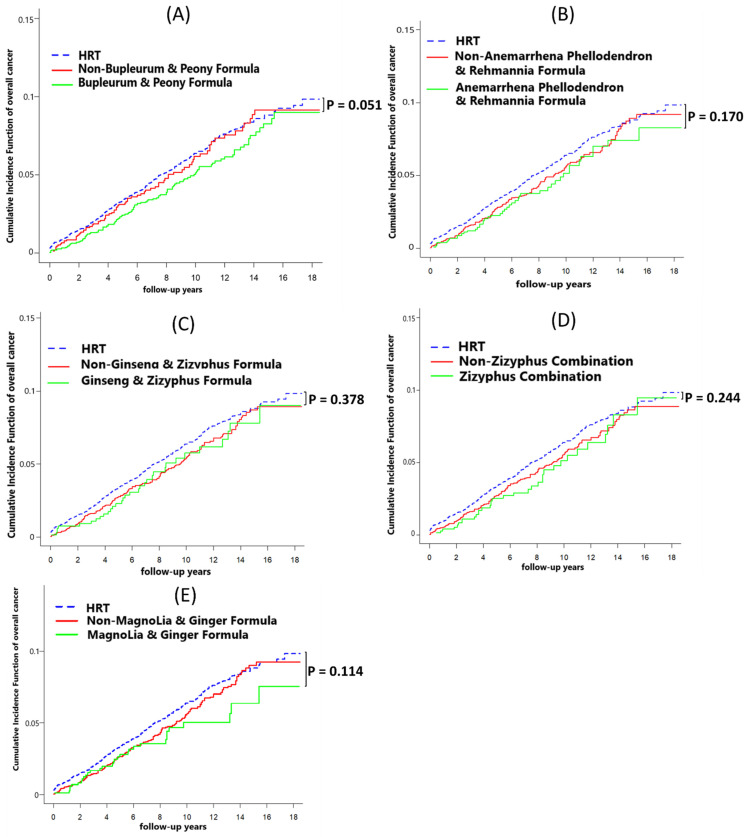
Cumulative incidence of overall cancer among the top five prescriptions of phytopharmaceuticals and HRT users after adjustment for competing mortality risks. (**A**) Bupleurum and Peony Formula. (**B**) Anemarrhena Phellodendron and Rehmannia Formula. (**C**) Ginseng and Zizyphus Formula. (**D**) Zizyphus Combination. (**E**) Magnolia and Ginger Formula.

**Figure 6:**
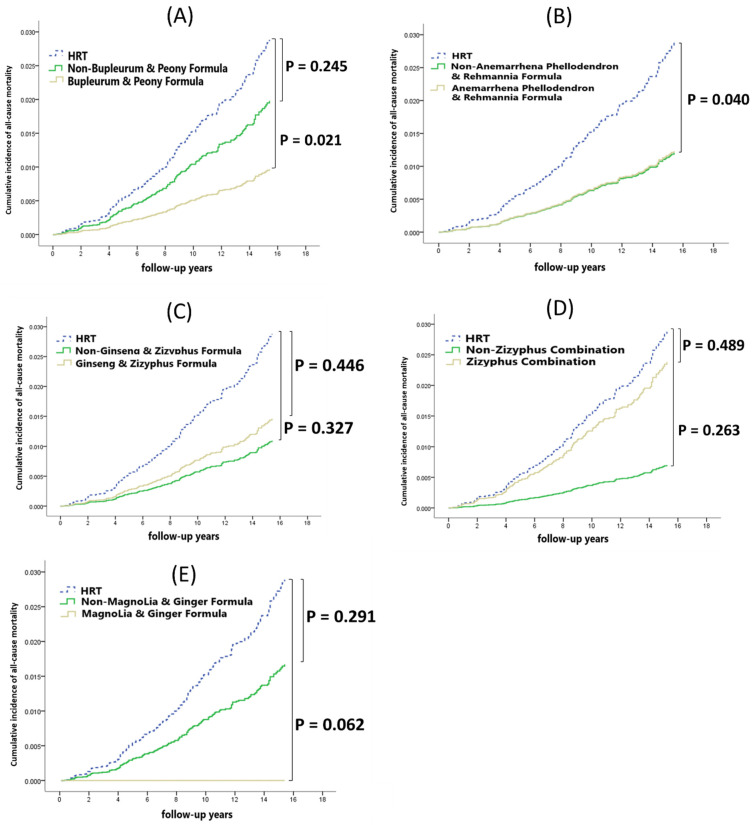
Cumulative incidence of all-cause mortality among the top five prescriptions of phytopharmaceuticals and HRT. (**A**) Bupleurum and Peony Formula. (**B**) Anemarrhena Phellodendron and Rehmannia Formula. (**C**) Ginseng and Zizyphus Formula. (**D**) Zizyphus Combination. (**E**) Magnolia and Ginger Formula.

The phytopharmaceutical users were divided into groups according to the top five prescriptions: BPF, APR, GZF, ZC, and MGF. After adjustment for potential confounders, we observed that some phytopharmaceutical prescriptions decreased the risks of overall cancer and mortality. For patients with BPF, the adjusted HR of overall cancer and all-cause mortality was 0.57 (95% CI: 0.36–0.92; *p* = 0.021) and 0.33 (95% CI: 0.11–1.05; *p* = 0.060) compared with that in the HRT group (Table 5). Compared to non-BPF patients and patients receiving HRT, the phytopharmaceuticals of BPF (*p* = 0.051) appeared to show a trend towards a decreased risk of overall cancer (Figure 5A). Furthermore, the phytopharmaceuticals of BPF (*p* = 0.021) were associated with all-cause mortality (Figure 6A).

## 4. Discussion

Our study is the first known to evaluate the risks of cancer and all-cause mortality between hormone replacement therapy (HRT) and phytopharmaceuticals in menopausal patients. Unlike previous research, this study employs a new-user design that ensures balanced baseline characteristics, allowing for a unique comparison of the progression of these outcomes.

### 4.1. New User Design for Balanced Characteristics in Groups

This study used a robust 1:2 new-user design with PSM without replacement to compare the outcomes of phytopharmaceuticals and HRT in menopausal patients. This design was employed to adjust for potential differences in demographics, comorbidities, and comedications between the two groups. To prevent immortal time bias, the study participants were assigned to treatment groups based on the date of their first prescription. Multivariate analysis was conducted to control for age, comorbidities, and CCI. Table 1 presents the balanced distributions of variables related to common comorbidities after matching.

### 4.2. Principal Outcomes

Although many studies have focused on the short-term effects of phytopharmaceuticals on vasomotor symptom relief, we explored the long-term risks of overall cancer and all-cause mortality associated with phytopharmaceuticals provided for managing menopausal syndrome [18,19].

Estrogen deficiency in menopausal women can lead to various long-term effects, including the development of osteoporosis, cardiovascular disease, and dementia; thus, HRT is a commonly used treatment. However, large, randomized trials, such as the WHI, have concluded that HRT is associated with increased risks of cardiovascular disease and invasive breast cancer across different racial/ethnic and age groups, and the risks are unaffected by prior disease status. Consequently, the global utilization of HRT has significantly declined [20,21].

Phytopharmaceuticals have been reported to be comparable to HRT in terms of effectiveness for treating menopausal hot flushes. Notably, herbal treatment alone did not decrease follicle-stimulating hormone levels, suggesting that the underlying mechanism of these medicinal herbs does not involve estrogenic effects and thus mitigating the concern regarding the phytoestrogenic properties of these herbs [22,23,24].

### 4.3. Endometrial and Cervical Cancer

Table 2 and Table 3 indicate that the incidence of each cancer was not significantly increased in the phytopharmaceuticals group compared with the HRT group. Notably, the phytopharmaceuticals group had a significantly low incidence of endometrial cancer and cervical cancer. The risks of endometrial cancer and cervical cancer were low, at 0.17 (95% CI: 0.07–0.38, *p* < 0.001) and 0.32 (95% CI: 0.14–0.71, *p* = 0.005), respectively. In Shen’s study, the proportion of patients with cervical cancer using phytopharmaceuticals was lower than that among those without cervical cancer (adjusted odds ratio: 0.8) [25]. These results strongly suggest that menopausal women in Taiwan should avoid the use of estrogen-only or estrogen-plus progestin and instead use phytopharmaceuticals for alleviating menopausal symptoms and potentially reducing the risks of endometrial cancer, cervical cancer, and other cancers. The underlying mechanism warrants further study.

### 4.4. Overall Cancer

Randomized trials have reported a potential association between HRT and cancer [26]. After an average follow-up of 5 years, WHI reported that patients on HRT have a 26% higher risk of cancer (HR: 1.26, 95% CI: 1.00–1.59). Specifically, the use of estrogen plus progestin for less than 5 years was associated with a 15% increase in cancer risk, while using it for more than 5 years was associated with a 53% increase [2]. The Heart and Estrogen/progestin Replacement Study also reported a 27% increase (nonsignificant) in the risk of cancer over 6.8 years of follow-up [27]. In a population-based study by Su et al., estrogen plus progestin use was associated with a 50% higher rate of breast cancer (HR: 1.48, 95% CI: 1.20–1.83) [28,29]. Our data indicated a 40% lower risk (HR: 0.60, 95% CI: 0.40–0.90; *p* = 0.015) of overall cancer in phytopharmaceuticals -only users with menopausal syndrome in Taiwan and over 180 days of use. Together, these findings suggest that cancer risk is reduced in menopausal women in Taiwan if they avoid the use of estrogen-only or estrogen plus progestin and use phytopharmaceuticals for menopausal symptoms. Further research is warranted to determine the underlying reasons.

### 4.5. All-Cause Mortality

The all-cause mortality outcome reflects the overall impact of therapy, considering the complex balance between benefits and risks. In Manson’s study from two WHI trials, the risk of all-cause mortality was 0.99 (0.94–1.03); for conjugated equine estrogen (CEE) plus medroxyprogesterone (MPA), the HR was 1.02 (0.96–1.08); and for CEE alone, the HR was 0.94 (0.88–1.01) [26]. In the study by Su et al., after a median follow-up duration of 110 months, the death rate was 0.92 (95% CI: 0.69–1.23) in the estrogen E-only group. However, in the estrogen + progestin group, the death rate was significantly lower than that in the unexposed group (HR: 0.79, 95% CI: 0.72–0.87) [27,29]. Many studies on HRT, such as the CEE plus MPA trial [2,30,31] or the CEE-only trial [30,32], have reported that during the intervention or cumulative follow-up, the rates of all-cause mortality were not elevated.

In our study (Table 2, Table 3 and Table 4), the risk of all-cause mortality of the phytopharmaceuticals group in Taiwan over 180 days of use was significantly lower (HR: 0.40, 95% CI 0.16–0.99; *p* = 0.047) than that in the HRT group. This finding supports the observation among Taiwanese menopausal women who use phytopharmaceuticals to manage their menopausal symptoms. Phytopharmaceuticals are not inferior to HRT in terms of their ability to decrease the risk of all-cause mortality.

### 4.6. Comparison between Subgroups of the Top Five Polyherbal Formulations

BPF is one of the representative formulas that harmonize liver stagnation and spleen deficiency, which are thought to cause mental or emotional problems, such as depression or anxiety [27]. BPF has been widely studied and found to improve haemorrheology, inhibit platelet aggregation, regulate the hypothalamic–pituitary–endocrine system, and affect immune-neuroendocrine function [24,33,34,35,36]. The study demonstrated that BPF treatment enhances memory in normal mice and improves memory deficits in mice with existing cognitive impairments. Additionally, BPF exhibits antidepressant effects by reducing levels of arginine vasopressin in the pituitary, decreasing the expression of arginine vasopressin mRNA in the hypothalamus [27] and increasing plasma tumor necrosis factor-α levels in menopausal patients with depression [37]. Among the five common prescriptions, only BPF was associated with a significant reduction in the risks of overall cancer and all-cause mortality, and other formulations yielded nonsignificant reductions.

Liu et al. reported that APR can regulate complement activation and inflammatory responses and promote antigen processing and presentation [38]. Studies have demonstrated that APF can ameliorate diabetic nephropathy symptoms by inhibiting glucose and lipid metabolism [39,40,41]. Although no studies on GZF, ZC, and MGF are available for reference, many reports exist on the main single ingredient of these formulations. The single herb in GZF, namely Rx Rehmanniae Conquitae (Shu-Dihuang), might possess therapeutic benefits for Alzheimer’s disease by regulating the expression of amy-1, sir-2.1, daf-16, sod-3, and hsp-16.2 [42]. The secondary metabolites of the single herb in ZC, namely Semen Zizyphi Spinosae (Suan-zao-ren), modulate GABAergic activity and the serotonergic system for the treatment of insomnia [43]. The single herb licorice, which is found in MGF, along with its natural compounds, demonstrates anti-inflammatory properties by reducing inflammatory mediators [44]. Although phytopharmaceuticals usually exert their effects in vivo through multicomponents, multiways, and multitargets, the underlying molecular mechanisms remain unclear [39,45]. Table 5, Figure 5 and Figure 6 show that compared with HRT, the five most common phytopharmaceutical prescriptions and the non-top five common phytopharmaceutical prescriptions were not associated with higher risks of overall cancer and all-cause mortality.

### 4.7. Limitations

Our study has several limitations. First, lifestyle factors, such as alcohol and cigarette consumption and body mass index, as well as laboratory test results, are not available in the NHIRD, which prevented us from assessing their impact on cancer risk and survival. Second, we could not distinguish the socioeconomic status and family history of the participants. Third, misclassification of ICD codes could have led to incorrect diagnoses of comorbidities. Fourth, we could not accurately measure compliance with phytopharmaceuticals and HRT medications by using data from the NHIRD, which may have affected prescription usage estimation. Fifth, information on over-the-counter prescription usage was limited, which could have underestimated exposure to nonprescription therapies. Finally, this observational study, based on NHIRD data, does not establish causal relationships. Additional evidence is needed to provide further support and clarification.

## 5. Conclusions

Our findings suggest that phytopharmaceuticals, particularly Bupleurum and Peony Formula, may be associated with lower risks of overall cancer and all-cause mortality compared to HRT, especially when used for more than 180 days. These results indicate that phytopharmaceuticals could serve as a safer alternative to HRT for alleviating menopausal symptoms while reducing the risks of cancer and mortality. Although estrogen and progestin have both advantages and disadvantages for menopausal women, they must be used with caution to reduce health risks and simultaneously provide health benefits. However, it is important to note that this observational study does not establish causal relationships, and further randomized controlled trials are necessary to validate these findings and explore the underlying mechanisms.

## Figures and Tables

**Table 1:** Baseline characteristics of the phytopharmaceuticals and HRT cohorts.

		Phytopharmaceuticals	%	HRT	%	*p*
Age	50–59	2452	60.9%	4904	60.9%	1.000
	60–69	1381	34.3%	2762	34.3%	
	>70	196	4.9%	392	4.9%	
CCI	0	1830	45.4%	3660	45.4%	1.000
	1–2	1445	35.9%	2890	35.9%	
	3–4	428	10.6%	856	10.6%	
	>5	326	8.1%	652	8.1%	
DM	No	3528	87.6%	7056	87.6%	1.000
	Yes	501	12.4%	1002	12.4%	
HCD	No	2881	71.5%	5762	71.5%	1.000
	Yes	1148	28.5%	2296	28.5%	
Hyperlipidemia	No	2828	70.2%	5656	70.2%	1.000
	Yes	1201	29.8%	2402	29.8%	
Statins	No	3115	77.3%	6184	76.7%	0.482
	Yes	914	22.7%	1874	23.3%	
Aspirin	No	3222	80.0%	5987	74.3%	0.000
	Yes	807	20.0%	2071	25.7%	
β-blocker	No	2240	55.6%	3605	44.7%	0.000
	Yes	1789	44.4%	4453	55.3%	
Metformin	No	3603	89.4%	7225	89.7%	0.689
	Yes	426	10.6%	833	10.3%	
Glucocorticoids	No	866	21.5%	1021	12.7%	0.000
	Yes	3163	78.5%	7037	87.3%	

Abbreviations used in this study: CCI—A measure of overall comorbidity burden, the Charlson Comorbidity Index. DM—Diagnosis of diabetes mellitus, HCD—Diagnosis of hypertensive cardiovascular disease.

**Table 2:** Incidence rates of each cancer between phytopharmaceuticals and HRT.

End Point	Phytopharmaceuticals	Incidence Rate *	(95% CI)	HRT	Incidence Rate *	(95% CI)
Ovarian cancer	24	0.68	0.46–1.02	44	0.63	0.47–0.85
Breast cancer	78	2.23	1.78–2.77	138	1.98	1.67–2.34
Endometrial cancer	6	0.17	0.08–0.38	67	0.96	0.76–1.22
Cervical cancer	7	0.20	0.09–0.42	50	0.72	0.54–0.95
Oral cancer	3	0.09	0.03–0.27	5	0.07	0.03–0.17
Thyroid cancer	12	0.34	0.19–0.60	17	0.24	0.15–0.39
Skin cancer	6	0.17	0.08–0.38	14	0.20	0.12–0.34
Colon cancer	15	0.43	0.26–0.71	37	0.53	0.38–0.73
Rectal cancer	12	0.34	0.19–0.60	21	0.30	0.20–0.46
Liver cancer	9	0.26	0.13–0.49	12	0.17	0.10–0.30
Lung cancer	21	0.60	0.39–0.92	36	0.52	0.37–0.72
Stomach cancer	4	0.11	0.04–0.30	8	0.11	0.06–0.23
pancreatic cancer	0	0.00	0.00–0.00	7	0.10	0.05–0.21
Kidney cancer	5	0.14	0.06–0.34	7	0.10	0.05–0.21
Bladder cancer	3	0.09	0.03–0.27	12	0.17	0.10–0.30
Overall cancer	197	5.62	4.89–6.46	452	6.48	5.91–7.10
All-cause mortality	75	2.14	1.71–2.68	173	2.41	2.07–2.79

* Incidence rate (per 1000 person-years).

**Table 3:** Adjusted hazard ratios for cancers and all-cause mortality comparing phytopharmaceutical and hormone replacement therapy cohorts.

End Point	Phytopharmaceuticals	%	HRT	%	*p*	HR (95% CI)	*p*
Ovarian cancer	24	0.6%	44	0.5%	0.394	1.39 (0.61–3.17)	0.437
Breast cancer	78	1.9%	138	1.7%	0.385	1.18 (0.89–1.57)	0.248
Endometrial cancer	6	0.1%	67	0.8%	0.000	0.17 (0.07–0.38)	0.000
Cervical cancer	7	0.2%	50	0.6%	0.000	0.32 (0.14–0.71)	0.005
Oral cancer	3	0.1%	5	0.1%	0.804	1.29 (0.30–5.55)	0.728
Thyroid cancer	12	0.3%	17	0.2%	0.366	1.42 (0.65–3.10)	0.380
Skin cancer	6	0.1%	14	0.2%	0.749	0.79 (0.30–2.10)	0.637
Colon cancer	15	0.4%	37	0.5%	0.486	0.75 (0.40–1.41)	0.379
Rectal cancer	12	0.3%	21	0.3%	0.714	0.92 (0.44–1.94)	0.825
Liver cancer	9	0.2%	12	0.1%	0.364	1.50 (0.61–3.69)	0.373
Lung cancer	21	0.5%	36	0.4%	0.576	1.29 (0.73–2.28)	0.381
Stomach cancer	4	0.1%	8	0.1%	1.000	1.08 (0.31–3.82)	0.901
pancreatic cancer	0	0.0%	7	0.1%	0.017	n/a	n/a
Kidney cancer	5	0.1%	7	0.1%	0.547	1.51 (0.46–4.93)	0.492
Bladder cancer	3	0.1%	12	0.1%	0.252	0.31 (0.07–1.44)	0.136
Overall cancers	197	4.9%	452	5.6%	0.095	0.87 (0.73–1.03)	0.111
All-cause mortality	75	1.9%	173	2.1%	0.293	0.91 (0.69–1.20)	0.519

n/a: According to the data protection policy of NHIRD, the events of the outcomes with phytopharmaceuticals <3 cases cannot be provided. HR adjusted for Age, CCI, DM, HCD, Hyperlipidemia, Statins, Aspirin, β-blocker, Metformin, and Glucocorticoids.

**Table 4:** Estimates for the association between phytopharmaceuticals use duration and risk of endpoints compared with HRT.

End Point	1–90 Days	*p*	91–180 Days	*p*	Over 180 Days	*p*
Ovarian cancer	1.75 (0.72–4.24)	0.214	1.62 (0.36–7.18)	0.527	n/a	-
Breast cancer	1.32 (0.96–1.82)	0.085	1.11 (0.63–1.97)	0.715	0.84 (0.44–1.60)	0.595
Endometrial cancer	0.17 (0.06–0.47)	0.001	0.30 (0.07–1.21)	0.091	n/a	-
Cervical cancer	0.28 (0.10–0.78)	0.015	0.26 (0.04–1.92)	0.188	0.48 (0.12–2.00)	0.316
Oral cancer	0.68 (0.08–5.88)	0.726	2.21 (0.25–19.4)	0.475	2.78 (0.32–24.5)	0.356
Thyroid cancer	1.81 (0.79–4.17)	0.162	0.76 (0.10–5.74)	0.786	0.71 (0.09–5.44)	0.745
Skin cancer	0.77 (0.25–2.38)	0.655	0.84 (0.11–6.47)	0.869	0.77 (0.10–5.96)	0.801
Colon cancer	0.97 (0.50–1.88)	0.918	0.30 (0.04–2.20)	0.237	0.32 (0.04–2.32)	0.257
Rectal cancer	0.75 (0.30–1.87)	0.532	1.23 (0.36–4.21)	0.737	1.00 (0.23–4.37)	0.999
Liver cancer	1.36 (0.46–4.02)	0.576	3.37 (1.04–10.9)	0.043	n/a	-
Lung cancer	1.42 (0.75–2.66)	0.280	0.72 (0.17–3.00)	0.649	1.23 (0.38–4.06)	0.729
Stomach cancer	1.23 (0.31–4.89)	0.773	n/a	-	1.45 (0.17–12.1)	0.732
Kidney cancer	1.67 (0.48–5.84)	0.422	1.46 (0.17–12.3)	0.726	n/a	-
Bladder cancer	0.49 (0.11–2.23)	0.357	n/a	-	n/a	-
Overall cancers	0.92 (0.75–1.11)	0.377	0.92 (0.66–1.29)	0.622	0.60 (0.40–0.90)	0.015
All-cause mortality	1.02 (0.75–1.38)	0.904	0.74 (0.42–1.31)	0.302	0.40 (0.16–0.99)	0.047

n/a: According to the data protection policy of NHIRD, the events of the outcomes with phytopharmaceuticals <3 cases cannot be provided. HR adjusted for Age, CCI, DM, HCD, Hyperlipidemia, Statins, Aspirin, β-blocker, Metformin, and Glucocorticoids.

**Table 5:** Associations of overall cancer and all-cause mortality among the top five prescriptions of phytopharmaceuticals and HRT.

	Overall Cancers	*p*	All-Cause Mortality	*p*
HRT (ref.)				
without BPF	0.65 (0.29–1.46)	0.294	0.68 (0.17–2.77)	0.594
BPF	0.57 (0.36–0.92)	0.021	0.33 (0.11–1.05)	0.060
without APR	0.56 (0.33–0.96)	0.034	0.41 (0.13–1.31)	0.133
with APR	0.63 (0.34–1.19)	0.153	0.42 (0.10–1.72)	0.229
without GZF	0.66 (0.41–1.06)	0.082	0.38 (0.12–1.18)	0.095
GZF	0.45 (0.20–1.01)	0.052	0.50 (0.12–2.04)	0.337
without ZC	0.58 (0.35–0.95)	0.031	0.24 (0.06–0.97)	0.046
ZC	0.62 (0.31–1.24)	0.176	0.83 (0.26–2.61)	0.744
without MGF	0.60 (0.37–0.97)	0.037	0.57 (0.23–1.41)	0.227
MGF	0.57 (0.27–1.21)	0.146	n/a	

Abbreviations: BPF, Bupleurum and Peony Formula; APR, Anemarrhena Phellodendron and Rehmannia Formula; GZF, Ginseng and Zizyphus Formula; ZC, Zizyphus Combination; MGF, MagnoLia and Ginger Formula. n/a: According to the data protection policy of the NHIRD, the events of the outcomes with phytopharmaceuticals <3 cases cannot be provided. HR adjusted for Age, CCI, DM, HCD, Hyperlipidemia, Statins, Aspirin, β-blocker, Metformin, and Glucocorticoids.

## Data Availability

The NHIRD has strict confidentiality guidelines in place to protect personal electronic data. Access to the research findings is limited to eligible researchers who meet the criteria for handling confidential data. Public sharing of these results is prohibited by the Taiwanese government under the “Personal Information Protection Act”. If you are interested in accessing the data, please feel free to submit a formal proposal to the National Health Insurance Research Database (NHIRD) for their review (https://dep.mohw.gov.tw/dos/np-2497-113.html, accessed on 26 July 2023). Alternatively, you can get in touch with the Taiwan Ministry of Health and Welfare’s staff using the provided contact information: Phone: 886-2-85906828; Email: sthuiying@mohw.gov.tw. Your efforts will be greatly appreciated.

## Supplementary Materials

The following supporting information can be downloaded at https://www.mdpi.com/article/10.3390/healthcare12121220/s1, Table S1: Functions and components of the five most commonly used herbal compound formulas for menopausal women.
