# Supplementary material for: Comparing Cancer Risks and Mortality between Phytopharmaceuticals and Estrogen-Progestogen Medications for Menopausal Women: A Population-Based Cohort Study
Source: Healthcare (Basel). 2024 Jun 19;12(12):1220. doi: 10.3390/healthcare12121220 (PMC11202969; doi:10.3390/healthcare12121220)
Supplement: Supplementary file 1 [file healthcare-12-01220-s001.zip › healthcare-3026007-supplementary.pdf]

Supplementary table 1. Functions and components of the five most commonly used herbal compound formulas for menopausal women

Name	Ingredients				Function	Reference
Bupleurum and	Radix Angelica Sinensis (Dang-	12.1%	Rx et Rz Glycyrrhizae	6%	Antidepressant effects	(1) Fan Xu et al., 2011
Peony Formula	Gui)		Prep cum Mel (Zhi-Gan-		through the influence on	[24]
	Rhizoma Atractylodis (Bai-Zhu)	12.1%	Cao)		central Arginine vasopressin	
(Jia Wey Shiau	Radix Paeoniae Alba (Bai-Shao)		Cortex Moutan (Mud-		systems.	
Yau Sann)	Radix Bupleuri (Cha-Ihu)	12.1%	Anpi)			
	Poriae (Fu-Ling)		Fructus Gardeniae (Shan-	7.57%		
			Zhizi)	7.57%		
		12.1%	Rhizoma Zingiberis (Wei-			
		12.1%	Jiang)	12.1%		
			Herba Menthae (Bo-He)	6%		
Anemarrhena	Rx Rehmanniae Conquitae (Shu-	27.5%	Cortex Moutan (Mu-Dan-		(1) Enhancement of antigen	(1) Liu H-R, et al.,2018 [33]
Phellodendron and	Dihuang)		Pi)	10.3%	processing and presentation	(2) Yokozawa et al., 2004 [35]
Rehmannia	Fructus Corni (Shan-Zhuyu)		Rhizoma Alismatis	10.3%	functions [35]	(3) Liu H-R et al.,2008 [36]
Formula	Poriae (Fu-Ling)	13.8%	(Ze-Xie)		(2) Renoprotective effects in	
(Zhi Bai Dih	Rhizoma Dioscoreae	10.3%	Rhizoma Anemarrhenae	6.9%	diabetic nephropathy (DN)	
Huang Wan)	(Shan-Yao)	13.8%	(Zhi-Mu)		[33][36]	
			Cortex Phellodendri	6.9%		
			Chinensis (Huang-Bai)			
Ginseng and	Radix Rehmanniae (Di-Huang)	6.25%	Radix Platycodonis (Jie-		Might possess therapeutic	Kang N et al., 2022 [37]
Zizyphus Formula	Rx Rehmanniae Conquitae (Shu-		Geng)	6.25%	benefits for Alzheimer's	
(Tian Wa Buu	Dihuang) (SD)	6.25%	Radix et Rhizoma Salvia		disease	
Shin Dan)	Radix et Rhizoma Ginseng (Ren-		iltiorrhizae (Dan-Shen)	6.25%		
	Shen)	6.25%	Semen Zizyphi Spinosae			
	Radix Polygalae (Yuan-Zhi)		(Suan-Zao-Ren)	6.25%		

	Rhizoma Acori Tatarinowii (Shi-Chang-Pu)	6.25%	Radix Ophiopogonis (Mai-Dong)	6.25%		
	Radix Asparagi (Tian-Men-Dong)	6.25%	Rhizoma Coptidis (Huang-Lian)	6.25%		
	Radix Scrophulariae (Xuan-Shen)	6.25%	Sclerotium Poriae Pararadicis (Fu-Shen)	6.25%		
	Semen Platycladi (Bai-zi-ren)	6.25%<br>6.25%	Radix Angelica Sinensis (Dang-Gui)	6.25%		
			Fructus Schisandrae Chinensis (Wu-Wei-Zi)	6.25%		
Zizyphus Combination (Suan Zao Ren Tang)	Semen Zizyphi Spinosae (Suan-Zao-Ren) (SZR)	41.7%	Rhizoma Anemarrhenae (Zhi-Mu)	16.6%	Modulating GABAergic activity and the serotonergic system for treating insomnia (SZR).	Shergis JL et al., 2017 [38]
	Rhizoma Chuanxiong (Chuan-Xiong)	16.6%<br>8%	Poriae (Fu-Ling)	16.6%		
	Rx et Rz Glycyrrhizae (Gan-Cao)					
**MagnoLia and Ginger Formula (Gan May Dah Tzao Tang)**	Rx et Rz Glycyrrhizae(Gan-Cao)	25%			Exhibiting anti-inflammatory properties (Gan-Cao).	Yang R et al., 2017 [39]
	Fructus Jujubae (Da-Zoa)	25%				
	Fructus Tritic i (Xiao-Mai)	50%				
